# The Effects of Fireworks Discharge on Atmospheric PM_2.5_ Concentration in the Chinese Lunar New Year

**DOI:** 10.3390/ijerph17249333

**Published:** 2020-12-13

**Authors:** Xuechen Zhang, Huanfeng Shen, Tongwen Li, Liangpei Zhang

**Affiliations:** 1 School of Resource and Environmental Sciences, Wuhan University, Wuhan 430079, China; zhangxc@whu.edu.cn; 2 State Key Laboratory of Information Engineering in Surveying, Mapping and Remote Sensing, Wuhan University, Wuhan 430079, China; zlp62@whu.edu.cn; 3 School of Geospatial Engineering and Science, Sun Yat-sen University, Zhuhai 519082, China; litw8@mail.sysu.edu.cn

**Keywords:** fireworks, PM_2.5_ concentration, Chinese Lunar New Year, remote sensing, firework prohibition policy

## Abstract

Discharging fireworks during the Chinese Lunar New Year celebrations is a deep-rooted custom in China. In this paper, we analyze the effect of this cultural activity on PM_2.5_ concentration using both ground observations and satellite data. By combining remote sensing data, the problem of uneven spatial distribution of ground monitoring has been compensated, and the research time span has been expanded. The results show that the extensive firework displays on New Year’s Eve lead to a remarkable increase in nationwide PM_2.5_ concentration, which were 159~223% of the average level, indicating the instantaneous effect far exceeds that of any other factor over the whole year. However, the averaged PM_2.5_ concentrations of the celebration period were 0.99~16.32 μg/m^3^ lower compared to the average values of the corresponding pre-celebration period and post-celebration period, indicating the sustained effect is not very significant. The implementation of firework prohibition policies can greatly reduce the instantaneous PM_2.5_ increase, but no obvious air quality improvement is observed over the entire celebration period. Combining these findings and the cultural significance of this activity, we recommend that this custom is actively maintained, using new technologies and scientific governance programs to minimize the negative effects.

## 1. Introduction

Discharging fireworks during the Chinese Lunar New Year is a custom that has continued for thousands of years. As early as the Northern and Southern Dynasties (420–589 AD), a book called “The Chronicle of Jingchu” had already recorded this custom [1]. According to the legend that a cannibal named Nian always attacked villages in winter. In the fight against Nian, people unexpectedly discovered that Nian did not like the color red and was afraid of flames and explosions, so they threw bamboo into the fire, and the fire plus the crackling sound successfully scared the monster away. Subsequently, this act of repelling the monster became a symbolic part of the New Year celebration. The word “Nian”, which has the same pronunciation as the word “year” in Chinese, has also become a synonym for “Chinese New Year”. Over time, with the development of gunpowder, fireworks and firecrackers gradually replaced bamboo and became the core element to the Chinese New Year celebrations.

However, despite the profound cultural significance of this traditional custom, the atmospheric pollution it causes has become a topic of national interest [2,3,4]. In recent years, China has faced unprecedented air pollution [5,6,7,8,9,10,11]. Among the many pollutants, PM_2.5_ (particles with an aerodynamic diameter of less than 2.5 μm) has received widespread attention due to its remarkable impact on haze events [12,13], local climate change [14], and especially human health [15,16,17,18]. It is now known that PM_2.5_ can be deposited deeply in the lungs through simple respiration, causing damage to the blood circulation system and the immune system [19], and it can induce respiratory and cardiovascular diseases [20,21]. In fact, outdoor PM_2.5_ pollution has become the number four risk factor responsible for the premature death of Chinese citizens [22]. Therefore, the task of controlling China’s PM_2.5_ level is very urgent.

Many activities can produce PM_2.5_, such as industrial production [23,24,25], transportation [26], heating [27,28], and agricultural waste treatment [29]. In recent years, high-intensity firework displays have attracted widespread attention because large amounts of harmful gases and particulate matter are generated when the fireworks are set off [30,31,32,33,34]. The pollutants produced by firework discharges not only seriously affect the surrounding air quality, but may also cause local haze [35,36]. The Independence Day firework displays in the U.S. have also been reported as causing a 42% increase of national 24-hr PM_2.5_, with an alarming 370% increase reported at an adjacent station [37]. During the night of the 2007–2008 Diwali Festival in India, it has been recorded that the 12-hr PM_2.5_ reached 591 μg/m^3^, which was almost 3.9 times the normal level [38]. During the Montreal International Fireworks Competition in 2007, it has been proven that PM_2.5_ remained above 1000 μg/m^3^ for almost 45 min within a 2-km-diameter area around the display site [39].

Due to the cultural meaning of fireworks in the Chinese New Year celebration, high-intensity displays are common, and the firework contribution to air pollution is considerable [40,41,42,43,44,45,46]. It has been reported that during the seven days of celebration following the Chinese Lunar New Year’s Eve in 2013, up to 5505 tons of firework residues were removed from the streets in Beijing [47]. Early in 2006, it has been reported that the firework displays contributed to sharp increase of both PM_2.5_ and PM_10_ [48]. Moreover, the study of Wuhan in 2014 showed the PM_2.5_ concentrations rise dramatically when massive firework display took place, and the pollution lasted for a few days [49]. Previous studies focusing on individual cities or specific city groups in China have indicated that fireworks discharge has a strong impact on PM_2.5_ concentration. However, most of the studies are highly targeted and therefore lack universality. In order to scientifically cognize and cope with the environmental effects of fireworks discharge in China, it is also necessary to conduct an in-depth and comprehensive assessment of the firework oriented PM_2.5_ pollution at a national scale. 

In this paper, the monitoring data of about 1600 stations (2013–2016) and 14 years of satellite remote sensing data (2002–2016) are used to systematically analyze the PM_2.5_ pollution caused by the fireworks discharge at New Year. Firstly, we evaluate the instantaneous effect of fireworks discharge based on the national daily maximum PM_2.5_ concentration, and we analyze the response degree of PM_2.5_ to fireworks in 31 provincial capital cities. As the Chinese New Year firework displays take place from New Year’s Day to the day after the Lantern Festival (Figure S1), we then investigate the sustained effect of these activities. Finally, we select some characteristic cities to evaluate the effect of firework prohibition regulations. By using all available ground observations, we tried to study the impact of fireworks on PM_2.5_ concentrations at a national scale, rather than a single city scale. With the help of historical ground PM_2.5_ data retrieved by satellite images, we have extended the research time range from four years to nearly fifteen years. Finally, by evaluating the effect power of fireworks on PM_2.5_ concentrations from multiple perspectives such as instantaneous effects, sustained effects and policy effects, we tried to draw a more comprehensive conclusion between fireworks and unconventional national PM_2.5_ pollution.

## 2. Materials and Methods 

### 2.1. Study Region and Data

The study region is China (Figure S2), and the research period is mainly from 18 January 2013, to 6 November 2016. Three kinds of data were used in this study:

(1) Ground PM_2.5_ observations. In recent years, the Chinese government has accelerated the construction of the PM_2.5_ ground monitoring network. By the end of 2016, the number of online sites had reached 1600. The collection and processing of pollutant data is undertaken in accordance with the Technical Regulation on Ambient Air Quality Index (AQI) and national quality control guidelines [50,51].
(2) Moderate Resolution Imaging Spectroradiometer (MODIS) aerosol optical depth (AOD) products. Compared with station observations, satellite data enjoy a wider coverage and longer observation times, which means that satellite data show significant advantages in air quality related research [52,53,54,55,56,57,58,59].
  In this study, MODIS Level 2 Collection 6 10-km AOD products were adopted for the national PM_2.5_ retrieval. The quality of MODIS AOD products has been proven to meet the requirements of atmospheric-related research [60,61,62]. The newly released 10-km AOD products in collection 6 have further enhanced the retrieval capacity in the highlighted regions by combining the dark target algorithm and deep blue algorithm in AOD retrieval. The overall correlation between Collection 6 AOD and AERONET-observed AOD over land is R = 0.86, and 69.4% of Collection 6 AOD fall within expected uncertainty of ±(0.05 + 12%) [63].
  The Terra and Aqua are polar-orbiting satellites and cross the equator at around 10:30 a.m. (descending orbit) and 1:30 p.m. (ascending orbit) local sun times, respectively. Since the crossing time of these two sensors is different, the data of Terra and Aqua should be combined to better represent daily AOD. For each pixel, if there is no data for both Terra and Aqua products, it was identified as “null”; if both values are identified, the average value was used; if there is only one valid value, this value was taken.
(3) Meteorological data. Meteorological conditions can greatly influence the distribution of atmospheric pollutants. The NCEP GDAS/FNL 0.25 Degree Global Tropospheric Analyses and Forecast Grids product is used in this research. These data have a 6-hr temporal resolution and fully cover the research area since 1991. We extracted relative humidity (RH, %), temperature (TEMP, K), wind speed, (WS, m/s), surface pressure (SP, Pa), and height of planetary boundary layer (HPBL, m) (Table S1) and resampled them into 0.1 degree (10 km) for historical PM_2.5_ retrieval. Because the spatial resolution of the reanalysis data was relatively coarse, the ground monitoring data was a better choice for the detailed analysis. The daily meteorological ground observations were also used to supplement the analysis of the firework policy effects.

### 2.2. Historical PM_2.5_ Retrieval

Ground observations are extensively used in surface air pollution. However, long-term research of China’s PM_2.5_ pollution has been severely restricted because of the late construction of the ground PM monitoring network. Besides, the distribution of ground stations is uneven. Thus, the remote sensing products have become popular in ground PM_2.5_ monitoring, because they can not only extend the research time, but also obtain the spatially continuous PM_2.5_ data [56,59,64]. 

Several attempts have been made to describe the AOD-PM relationship, and most of them can be classified into simulation-based models or observation-based models [65]. Because the requirement of datasets is more comprehensive for simulation-based models (especially emission inventories can be hard to acquire sometimes) [64,66], the observation-based models can be a good compromise [67]. 

According to previous researches [68,69,70,71], the AOD-PM relationship has gradually been regarded as a nonlinear problem of multiple variables. Therefore, artificial neural networks, which can better present complex nonlinear relationships, have been used to estimate PM_2.5_ concentrations [67,72,73]. A three-layer back-propagation neural network (BPNN) model was constructed to estimate historical daily PM_2.5_ [67]. 

**Figure 1:**
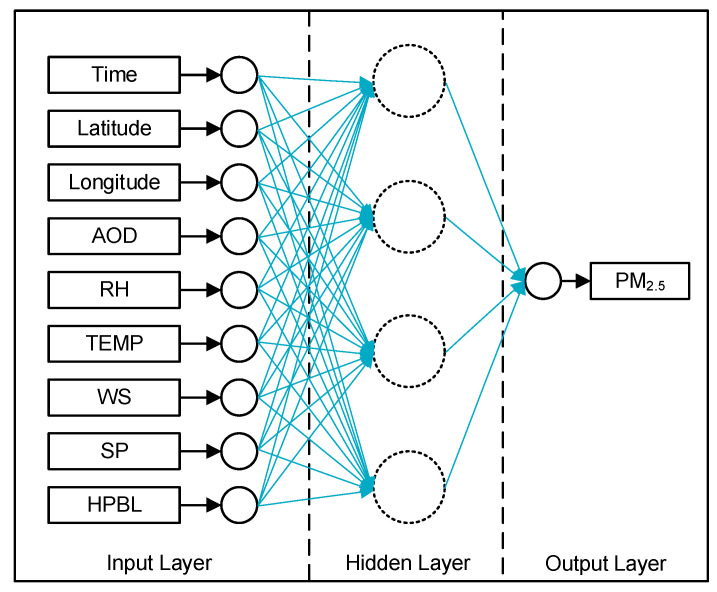
The structure of neural network model.

It takes a total of nine factors as input, including spatiotemporal information, AOD, and meteorological elements, and gives PM_2.5_ as the only output, the model structure is shown in Figure 1. The optimal performance node number of the hidden layer is usually between 2+μ and 2n+1 (where n and μ represent the number of nodes in the input layer and the output layer, respectively) [74]. In previous work, 18 nodes were proved to have the best performance [75].

The model performance was evaluated using the correlation coefficient (R) and root-mean-square error (RMSE) between the measured PM_2.5_ and the retrieved PM_2.5_, and 10-fold cross-validation was introduced to assess the possibility of model overfitting [76]. The formulas of R and RMSE are defined as follows:

(1) $$R=\frac{\sum_{i=1}^{n}\left(y_{i}-y^{*}\right)\left(y_{i}^{\prime }-y^{*}\right)}{\sqrt{\sum_{i=1}^{n}{\left(y_{i}-y_{i}^{\prime }\right)}^{2}\sum_{i=1}^{n}{\left(y_{i}^{\prime }-y^{*}\right)}^{2}}}$$

(2) $$\mathrm{RMSE}=\sqrt{\frac{1}{n}\overset{n}{\underset{i=1}{\sum }}{\left(y_{i}-y_{i}^{\prime }\right)}^{2}}$$

where n describes the sample size; $y_{i}$ and $y_{i}^{\prime }$ are the observation and prediction values of the PM_2.5_ concentrations, respectively; $y^{*}$ is the average value of PM_2.5_ observations.

The R describes the fitting degree of the constructed model, and usually the higher value indicates the better model reliability. The RMSE describes the prediction error of the model, and usually the lower value indicated the better model accuracy. As for the 10-fold cross-validation method, the sample-based cross-validation is adopted to evaluate the overall predictive performance of satellite-based PM_2.5_ retrieval models [77]. In sample-based cross-validation, all the samples are almost equally divided into 10 folds randomly, and the complete validation needs 10 rounds. Each round, nine folds will be used for model fitting and the remaining one will be used for model validation.

**Figure 2:**
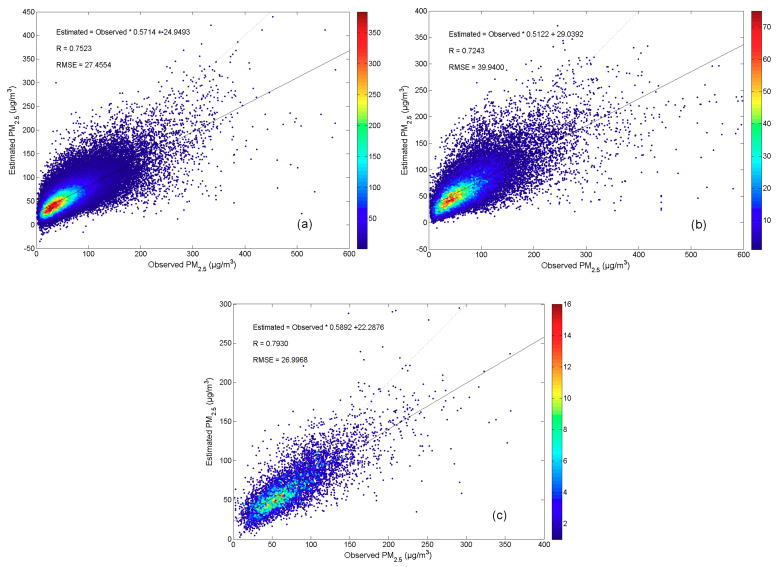
(**a**) Scatter maps of model cross-validation result, (**b**) model prediction result, and (**c**) model 17-day prediction result.

In this work, the main purpose of remote sensing data retrieval is to obtain historical ground PM_2.5_ data, so as to explore the influence of the Chinese New Year fireworks on PM_2.5_ concentrations in a longer time span. Thus, we used the data from 1 March 2014 to 29 February 2016 to construct the model, to estimate the data from 1 March 2013 to 28 February 2014. The model fitting R is 0.7581 and RMSE is 27.1316 μg/m^3^, the model cross-validation R is 0.7523 and RMSE is 27.4554 μg/m^3^ (see Figure 2a). These values indicated that the model is not seriously overfitting. The model prediction R is 0.7243 and RMSE is 39.9400 μg/m^3^ (see Figure 2b), and the model 17-day prediction R is 0.7930 and RMSE is 26.9968 μg/m^3^ (see Figure 2c). These values indicated that the model can be considered robust for historical PM_2.5_ retrieval, especially for the latter historical 17-day fusion research. Furthermore, we retrieved PM_2.5_ data from 2002 to 2012 for the evaluation of fireworks’ long-term effect on PM_2.5_ concentrations.

### 2.3. The 17-Day Time Division Schema

**Figure 3:**
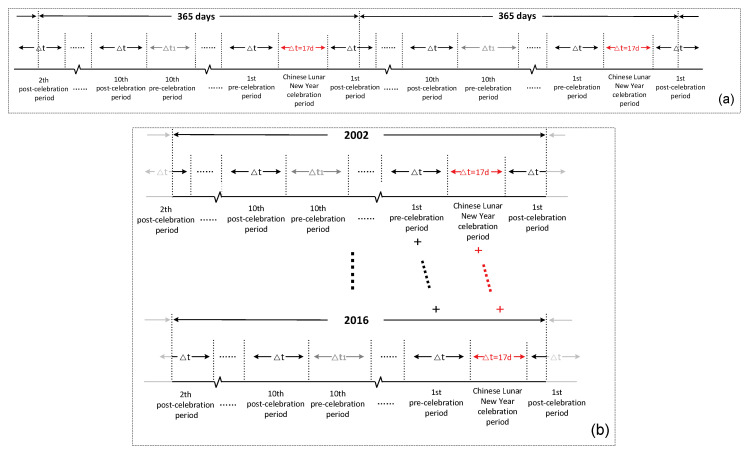
(**a**) The 17-day time division schema and (**b**) the historical PM_2.5_ data fusion schema.

In this research, we consider the entire Chinese New Year celebration period as a whole and denote its time span (17 days) as Δt. Using Δt as division unit, and taking each celebration period as an anchor point, we were able to divide the time before and after each celebration period. As the result, the time from 2002 to 2016 can be circularly divided into twenty-one periods in sequence (the schematic diagram is shown in Figure 3a). The order of periods is: the 10th pre-celebration period~ the 1st pre-celebration period, the celebration period, and the 1st post-celebration period~ the 10th post-celebration period.

Since the date of the Chinese New Year was different every year (see Table S2), after divided the 9th periods, the number of days left in each year would be slightly different. These days were mainly in the summer and had little effect on the study of the Chinese New Year celebration periods in winter. Therefore, we still divided the 10th post-celebration period by Δt and put the rest of the days into the 10th pre-celebration period. Thus, each twenty-one period is composed of twenty 17-day periods and one residual period (the 10th pre-celebration period).

### 2.4. The Historical PM_2.5_ Data Fusion Schema

As mentioned above, ground observations may have some deficiencies in related researches due to the construction time and location constraints of the stations. At present, the satellite retrieved PM_2.5_ data are generally considered to be good supplementary data. However, constrained by imaging conditions, such as bright ground, clouds, and fog, most satellite products are troubled by data missing. Thus, the retrieved PM_2.5_ data usually cannot achieve full daily coverage. Therefore, it is necessary to adopt appropriate fusion strategies to improve data coverage before further analysis. 

Through the 17-day time division schema, the division results can be illustrated as Figure 3b. To compare the PM_2.5_ levels between different time periods across these years, the retrieved data of the same period from fifteen years were fused. For example, the data of every Chinese New Year celebration period in 2002–2016 were fused into one distribution map of PM_2.5_ concentrations (illustrated in Figure 3b with red letters). The data of other periods were fused with the same strategy.

## 3. Results

### 3.1. Instantaneous Effect of Fireworks Discharge on PM_2.5_

Based on all the available ground monitoring data from January 18, 2013, to November 6, 2016 (including the four celebration periods, see Table S3), we obtained continuous hourly average PM_2.5_ concentrations for mainland China. 

**Figure 4:**
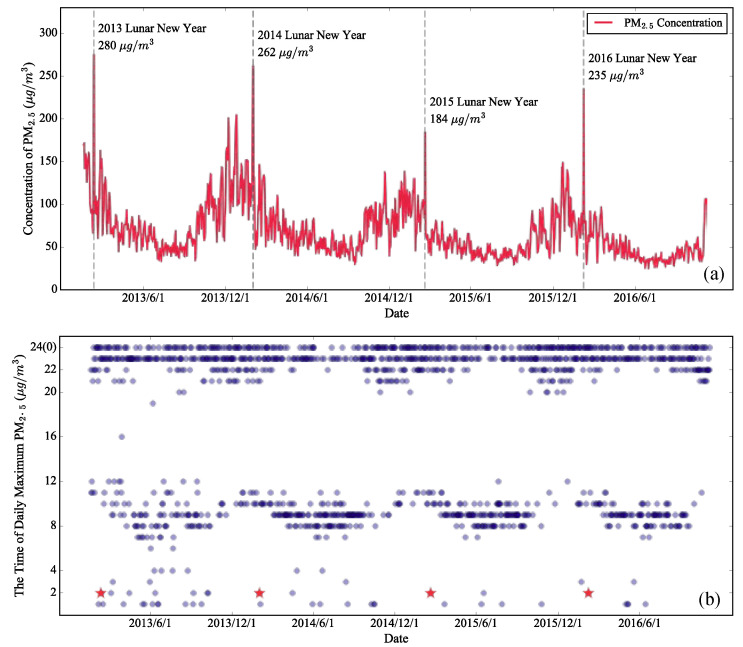
(**a**) Station-based daily maximum PM_2.5_, and (**b**) its time distribution in mainland China from 18 January 2013, to 6 November 2016. Each New Year’s Day is marked with a dashed gray line (in (**a**)) or a red pentagram (in (**b**)), and the corresponding PM_2.5_ concentrations are given in (**a**).

Firstly, we recorded the daily maximum PM_2.5_ (Figure 4a). It can be seen that the daily maximum PM_2.5_ reaches its lowest point in summer and its highest point in winter, with a clear pattern of seasonal variation [55,78]. The severe PM_2.5_ pollution in winter is likely the result of winter heating [27,28] and stable meteorological conditions [79,80].

Remarkably, the most significant increase appears on the Lunar New Year in all four years (i.e., 10 February 2013; 31 January 2014; 19 February 2015; and 8 February 2016 in the solar calendar). The corresponding PM_2.5_ levels on these days reached 280 μg/m^3^, 262 μg/m^3^, 184 μg/m^3^, and 235 μg/m^3^, which were, respectively, 209%, 159%, 223%, and 206% of the average concentrations of the days before and after the Lunar New Year.

It should be noted that these are the highest values of the corresponding years, and these peaks all appeared at 2 a.m. on Lunar New Year’s Day. So, we recorded the time when the national average PM_2.5_ reached the maximum each day to observe its distribution. According to Figure 4b, daily PM_2.5_ usually reached a maximum around 7:00–11:00 or 21:00–24:00 (00:00). Of the 1389 days considered in this study, only 12 maximum daily PM_2.5_ concentrations appeared at 2 a.m., and the PM_2.5_ concentrations of the other eight days were far below the PM_2.5_ level of each Lunar New Year (i.e., 94 μg/m^3^, 61 μg/m^3^, 62 μg/m^3^, 40 μg/m^3^, 99 μg/m^3^, 72 μg/m^3^, 69 μg/m^3^, and 38 μg/m^3^, respectively). 

Lunar New Year’s Eve and Lunar New Year’s Day are the time when most intensive fireworks discharge takes place. In terms of time, these New Year peaks are most likely caused by fireworks. Meanwhile, the closure of many factories and enterprises has greatly suppressed the PM_2.5_ pollutants emitted by economic activities [45,81]. Under such favorable conditions, the daily maximum PM_2.5_ still reaches extremely high levels on the Lunar New Year, indicating that the Chinese Lunar New Year fireworks discharge has the strongest transient influence in the whole year. These conclusions confirmed some city-level conclusions on a national scale.

Although we had confirmed that the effect of fireworks discharge is serious nationwide, we still wanted to check if this effect was equally significant in individual cities. We therefore decided to conduct an analysis in 31 provincial capital cities, including the capital Beijing, because ground data are usually more widely available in these cities. Because fireworks seem to have the greatest impact on PM_2.5_ in the morning of New Year’s Day (Figure S3), and PM_2.5_ tends to reach its highest level at 2 a.m., we decided to explore the response of PM_2.5_ to fireworks based on the time window of 00:00–04:00. During each celebration period, each city was categorized into one category (significant response, obvious response, or weak response), according to the response of PM_2.5_ to fireworks discharge.

The classification was conducted as follows:

(1) If the maximum value of PM_2.5_ in a city appeared during 0:00–04:00 on New Year’s Day, it was considered that the city’s PM_2.5_ responded significantly to the New Year fireworks.
(2) If the maximum value of PM_2.5_ in a city did not appear during 0:00–04:00 on New Year’s Day, but both the increase and the increase rate were the highest compared with those during the 0:00–04:00 period of the remaining days, it was considered that the city’s PM_2.5_ responded obviously to the New Year fireworks.
(3) If the variation of PM_2.5_ in a city did not match the above two conditions, it was considered that the city’s PM_2.5_ responded weakly to the New Year fireworks.

To avoid any possible interference, the data from the Lantern Festival period were excluded from this classification. In addition, according to the Tibetan calendar (Table S4) and related customs, the data from the Tibetan New Year firework period would not affect the classification results. 

**Figure 5:**
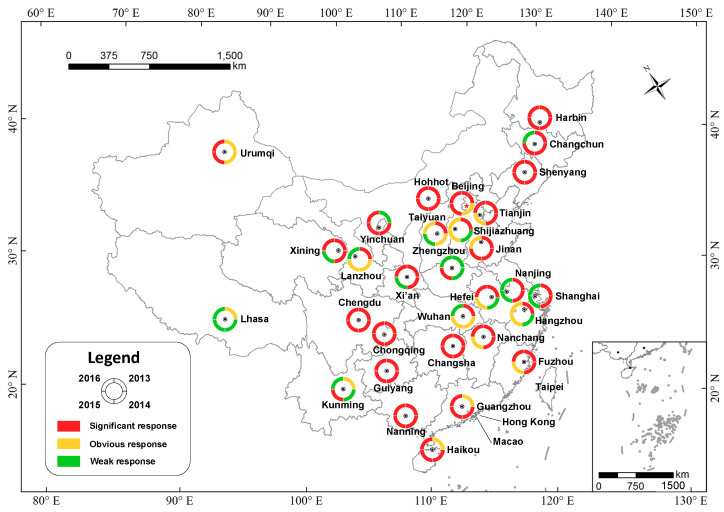
PM_2.5_ response to fireworks discharge in 31 provincial capital cities during the 2013–2016 New Year celebration periods.

According to the classification map shown in Figure 5 and the corresponding statistical results (Table S5), it is unsurprising to see that the cites where the PM_2.5_ showed a significant response to fireworks were in the majority during the 2013–2016 celebration periods in the Chinese Lunar New Year, amounting to 74.2%, 61.3%, 51.6%, and 58.1% of the total, respectively. In contrast, the proportion of cities with a weak response was almost the lowest, amounting to 6.5%, 9.7%, 19.4%, and 25.8%, respectively. Notably, only two cities fell into the weak response category in 2013. Even taking different diffusion conditions into account, it is still reasonable to say that the discharge of Chinese Lunar New Year fireworks is causing significant instantaneous PM_2.5_ increases. More importantly, such a phenomenon does not only occur in individual cities, but in 31 cities that are across the whole country.

### 3.2. Sustained Effect of Fireworks Discharge on PM_2.5_

**Figure 6:**
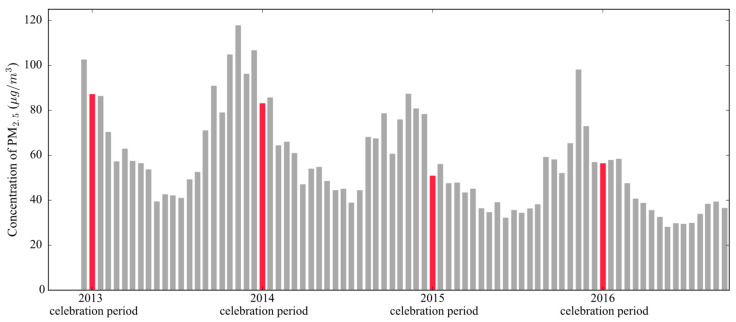
The 17-day averaged PM_2.5_ concentrations from 2013 to 2016. The red bars represent the average PM_2.5_ values during the four New Year celebration periods.

Since the transient effect is so strong, we could not help but wonder how strong the sustained effect is. As can be seen in Figure 4a and Figure S3, although the PM_2.5_ on New Year’s Day showed a fairly sharp increase, the PM_2.5_ concentrations dropped back to the usual level over the next few days. In fact, there is usually a great variety of spontaneous celebrations and commemorations over the entire 17-day celebration period, which often involve the discharge of fireworks. Therefore, it was necessary to explore the continuous influence of New Year’s fireworks on PM_2.5_ throughout the entire celebration period. To undertake this analysis, each Chinese lunar year was divided into 21 periods by the same time span as the celebration period through the 17-day interval division scheme. The average national PM_2.5_ monitoring values during each period in 2013–2016 were calculated and are shown in Figure 6.

As can be seen, the phased concentrations of PM_2.5_ is in accordance with the seasonal variation appeared in Figure 4, and interestingly, even under the dramatic transient effect of the fireworks, the celebration period PM_2.5_ concentration does not break the seasonal variation pattern. The PM_2.5_ of four celebration periods were 87.43 μg/m^3^, 83.37 μg/m^3^, 51.13 μg/m^3^, and 56.68 μg/m^3^, respectively. The corresponding PM_2.5_ averages of pre- and post-celebration periods were 94.71 μg/m^3^, 96.46 μg/m^3^, 67.45 μg/m^3^, and 57.68 μg/m^3^, respectively. The PM_2.5_ in four celebrations were all below the average level of pre- and post-celebration periods, and the PM_2.5_ level was even lower than that of both the pre- and post-celebration periods in 2014, 2015, and 2016. In addition, although the PM_2.5_ level during the 2013 celebration period was slightly higher than that of the post-celebration period, it was still much lower than that of the pre-celebration period. It therefore seems that the sustained effect of the New Year fireworks is not as strong as the instantaneous effect.

**Figure 7:**
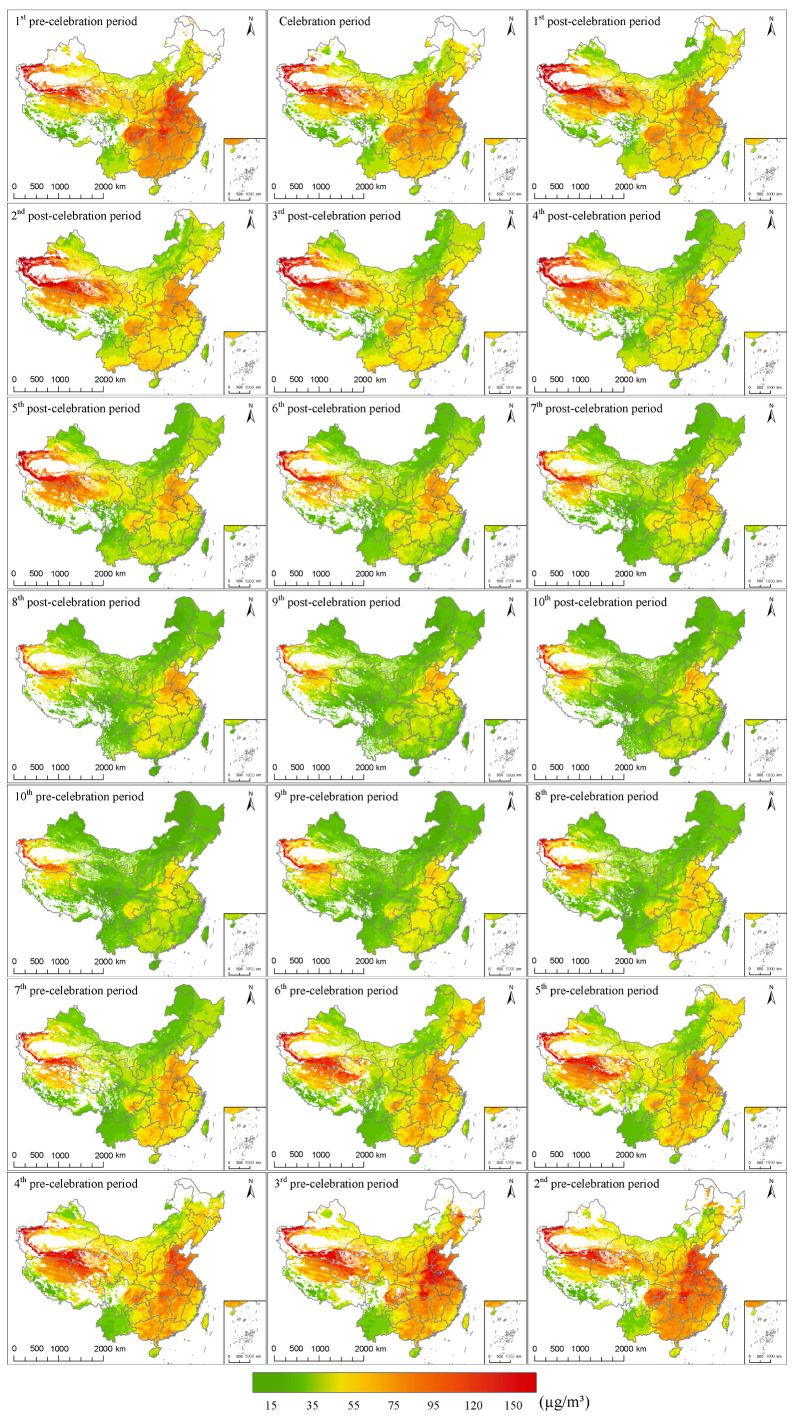
Fused PM_2.5_ distribution maps for mainland China from 2002 to 2016. The fusion maps are basically arranged in chronological order.

To further establish the sustained effect of New Year’s fireworks over a longer period of time, Terra and Aqua Moderate Resolution Imaging Spectroradiometer (MODIS) satellite data were employed. We used the 10-km AOD products to retrieve the near-ground PM_2.5_ data from 2002 to 2016, and further combined this with the 17-day interval division scheme to obtain fusion maps of PM_2.5_ distribution in mainland China for 2002–2016 (Figure 7).

From Figure 7, we can see the retrieved PM_2.5_ showed similar spatial distributions to the ground observations. According to previous studies [82], the PM_2.5_ level in northern China was generally higher, and the PM_2.5_ level in the southern area was relatively lower, which is mainly affected by population distribution and urbanization progress. 

As is known to us all, the celebration period is usually in the middle of winter when PM_2.5_ pollution is at its worst. As in Figure 7 we can see that the PM_2.5_ does show a significant pattern of seasonal variation, it decreases during the spring (from the 1st post-celebration period to the 5th post-celebration period), and reaches the lowest point in the summer (from the 6th post- celebration period to the 10th pre-celebration period), then gradually increases through the autumn (from the 9th pre-celebration period to the 5th pre-celebration period), and finally reaches the highest point in the winter (from the 4th pre-celebration period to the celebration period). The regional aggregations indicated that there are many other factors that play important roles in PM_2.5_ accumulation. For example, the PM pollution in Xinjiang is possibly due to the existence of deserts, while the PM_2.5_ hot spots in Sichuan and Beijing-Tianjin-Hebei region are not only affected by population density, but also because of the surrounding mountains make it difficult for the pollutants to spread [83].

As for smaller time window, compared with the pre-celebration period, PM_2.5_ in the celebration period shows a remarkable decrease, especially in areas with higher PM_2.5_ concentrations, such as the Beijing-Tianjin-Hebei region and the Sichuan Basin. In the post-celebration period, the pollution is further alleviated, which is more prominent in central and eastern China. Although the station-based overall PM_2.5_ level of the celebration period shows a gradual decreasing trend which is consistent with its seasonal variation, it is still unexpected to see that the PM_2.5_ of the entire celebration period shows no obvious increase across the country.

### 3.3. Effect of Firework Prohibition Policies

Since the debate is still fierce over whether to ban fireworks discharge to improve air quality, some local governments have attempted to implement such policies in China as attempts [84]. As the statistical results showed in Table S5, the number of cities with a weak response showed a certain increase throughout 2013–2016, which is very likely to be a policy-oriented phenomenon. It is therefore necessary to analyze and evaluate the effects of these policies.

Considering many factors, we decided to take the cities of Wuhan, Chengdu, and Zhengzhou as examples. These cities implemented firework prohibition policies in 2014, 2015, and 2016, respectively, which makes the data of these cities very suitable for comparing the changes in PM_2.5_ levels before and after the policies’ implementation.

The 34-day mean PM_2.5_ concentration during the pre-celebration period (17 days) and the post-celebration period (17 days) were used to represent the normal PM_2.5_ level, and the difference between the hourly PM_2.5_ concentrations and the corresponding averages was then calculated to indicate the policy efficiency. 

**Figure 8:**
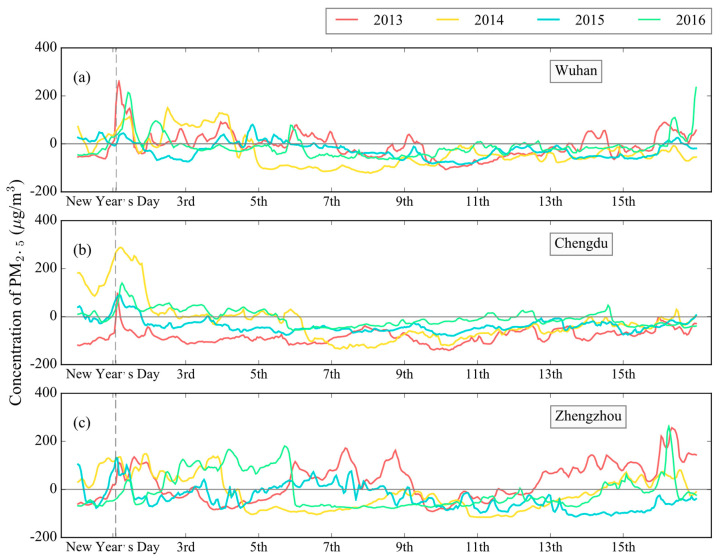
The difference between celebration period hourly PM_2.5_ concentrations and the corresponding 34-day average PM_2.5_ concentrations for 2013–2016 in (**a**) Wuhan, (**b**) Chengdu, and (**c**) Zhengzhou. The gray dotted line marks 2 a.m. on New Year’s Day.

From Figure 8, we can see when focusing on the variation of PM_2.5_ pollution in the Chinese Lunar New Year’s Eve and the early morning of New Year’s Day, it is clear that the implementation of the firework prohibition policies significantly reduced the instantaneous PM_2.5_ increase. For example, PM_2.5_ concentration sharply increased in Wuhan on New Year’s Day in 2013, but after the implementation of the prohibition policy in 2014, the PM_2.5_ concentration decreased significantly in the next three celebration periods. For Chengdu, the PM_2.5_ concentrations in the last two years, under the prohibition policy, were also lower than those of the previous two years, despite the remaining visible peaks. As for Zhengzhou, the PM_2.5_ concentrations were all very high in 2013–2015, but the contamination level was greatly reduced in 2016 when the prohibition policy took effect. 

However, when analyzing the whole of the Chinese Lunar New Year celebration period, it is difficult to say if the prohibition policy is still that effective. This is likely because other factors such as meteorological conditions and daily emissions can also profoundly influence the pollution level of PM_2.5_. For example, the purification effect of heavy precipitation made the air much cleaner in 2015 than in 2014 and 2016 in Wuhan, although prohibition policies were in effect in all these three years (see Table S6). In Chengdu in 2013, although the fireworks discharge significantly increased the instantaneous PM_2.5_ concentrations, the favorable weather conditions still made the PM_2.5_ level in the few days around the New Year’s Day lower than those in the years when the prohibition policies were implemented (see Table S7). In contrast, in 2014, the stable meteorological conditions in Chengdu greatly exacerbated the accumulation of pollutants, resulting in this city suffering from much more severe PM_2.5_ pollution [85]. Another example about industrial and municipal discharges is in 2016, the New Year peak of PM_2.5_ in Zhengzhou was noticeably eased under the implementation of the prohibition policy, but under the same weather conditions (see Table S8), the PM_2.5_ pollution situation began to deteriorate from the second day of the new year, and then the serious pollution lasted for four days. This phenomenon was probably because daily emissions were still causing a continual PM_2.5_ increase, and the reduction of pollutants by firework discharge regulations were not effective enough. Similarly, the effect of daily pollution can also be observed in Wuhan and Chengdu from 2013 to 2016. For most of the time after the seventh day of the Lunar New Year, the PM_2.5_ was lower than the normal level. It is especially apparent for Chengdu in 2013, where the PM_2.5_ level was almost always lower than usual, except for the early hours of New Year’s Day.

## 4. Discussion

The results of this study indicate that the intense discharge of fireworks at the Chinese New Year can dramatically increase PM_2.5_ over a short period of time, and this effect exceeds that of any other factor nationwide. However, the sustained effect of the fireworks is not strong. On the one hand, the discharge of fireworks at New Year is intense, but the duration is short, and when combined with certain meteorological conditions [86], it allows the PM_2.5_ to diffuse and settle quite quickly in the relatively less-polluted surroundings. On the other hand, during the celebration period, some major sources of daily PM_2.5_ pollution, such as factory and traffic emissions, are greatly reduced due to factory shutdown, festive population migration, and other related factors. Thus, the “holiday effect” may also play an important role in curbing PM_2.5_ pollution [45,81]. Therefore, it is rational to believe that the aggravation of PM_2.5_ pollution caused by the Chinese Lunar New Year fireworks discharge is no stronger than the mitigation brought by the natural cleaning processes and the “holiday effect”.

As for the effectiveness of firework prohibition policies, it is clear that these policies can significantly alleviate the PM_2.5_ bursts caused by fireworks discharge in the early hours of Lunar New Year’s Day. However, we have to admit that its influence has limitation over the whole celebration period, which is probably because there are still many other strong influence factors, such as meteorological conditions and daily emissions, that can offset or even overshadow the effect of policies. Additionally, it is important to take the cultural and symbolic meaning of Chinese Lunar New Year fireworks into account, in that people may still want to celebrate the Chinese Lunar New Year in the traditional way, no matter what [87]. During 2014 and 2015 in the Chinese Lunar New Year celebration periods in Chengdu, the PM_2.5_ level still showed an obvious increase in the early morning of New Year’s Day, despite the prohibition policy being in effect. Additionally, during the 2014 celebration period in Wuhan, even if they may get punished, people still celebrated the New Year in the traditional way [88].

Firework prohibition policies can indeed alleviate the transient pollution, but their effect on the sustained PM_2.5_ concentration during the entire celebration period of the Chinese Lunar New Year is not obvious. As a Chinese culture passed down for thousands of years, the custom of discharging fireworks during the Chinese Lunar New Year is deeply rooted. It therefore seems overly simplistic and unreasonable to completely prohibit this celebration activity only for the air pollution consideration. More appropriate approaches should be adopted in a more humanized and scientific way. For example, the government could consider guiding the public to rationally discharge fireworks at appropriate times and places, according to diffusion conditions and safety factors. Alternatively, we could encourage the development of low-pollution substitutes, including replacing the traditional high-polluting gunpowder with environmentally friendly desulfurized explosives, and adopting electronic fireworks products.

## 5. Conclusions

To date, there have been some studies of PM_2.5_ variation with firework displays, but most of the works have focused on individual cities [40,42,45,48,49,89]. However, considering that China has a large span of latitude and longitude, the climatic characteristics and geographical conditions are quite different, which makes the researches based on a single city have certain limitations. Therefore, a comprehensive analysis of the effect of Chinese Lunar New Year fireworks discharge on PM_2.5_ concentration in China was necessary.

In this study, we jointly used station monitoring data and remote sensing products to comprehensively analyze the effects of the Chinese Lunar New Year fireworks discharge on PM_2.5_ concentration within the entire mainland China. By using remote sensing data, the problem of uneven spatial distribution of ground monitoring has been compensated, and the research time span has been expanded from four years (ground observation time) to fifteen years (satellite observation time).

The results indicate that the intensive Chinese Lunar New Year fireworks discharge can contribute to a sharp increase of PM_2.5_ within a short period of time. Compared with the concentrations before and after each Lunar New Year, the PM_2.5_ of the four Lunar New Years increased by 109%, 59%, 123%, and 106%, respectively. In most cities (ranging from 51.6~74.2%), PM_2.5_ variations during the Lunar New Year showed strong correlations with the discharge of fireworks. However, due to the suitable diffusion conditions and the reduction of factory and traffic emissions, the overall PM_2.5_ concentration during the 17-day celebration period of the Chinese Lunar New Year is no higher, and even lower than the normal level (which were 7.28 μg/m^3^, 13.09 μg/m^3^, 16.32 μg/m^3^, and 0.99 μg/m^3^ lower compared to normal level, respectively). Therefore, the sustained effect of fireworks discharge on PM_2.5_ concentration is not as serious as many people may once have thought. As for the firework prohibition policies, its implementation has been proved to have a significant effect on the reduction of the instantaneous PM_2.5_ peaks. The PM_2.5_ increase during the concentrated fireworks discharge hours (00:00–04:00 on Lunar New Year’s Day) has been significantly reduced. However, its impact on the entire celebration period is limited. The PM_2.5_ levels did not show any significant changes during the rest of the celebration period before and after the implementation of the policies. At the same time, other factors may also have a certain impact on the effect of policy implementation.

The custom of setting off fireworks during the Chinese Lunar New Year has been passed down for thousands of years, and it seems too arbitrary to completely abolish this custom only for air pollution consideration. We believe that this significant custom should be continued with minimized negative effects, with the assistance of new technology and scientific management.

## Supplementary Materials

The following are available online at https://www.mdpi.com/1660-4601/17/24/9333/s1, Figure S1. Scheme of important timing during the Chinese Lunar New Year celebration period; Figure S2. Distribution of PM_2.5_ monitoring stations in mainland China by the end of 2016; Figure S3. National hourly PM_2.5_ concentration during New Year’s Eve and the next two days; Table S1. Meteorological data information; Table S2. Chinese New Year dates in 2002–2016; Table S3. Dates of important days during 2013–2016 Chinese Lunar New Year celebration periods; Table S4. Tibetan New Year date and its firework display time; Table S5. Classification results of PM_2.5_ response in 31 provincial capitals during 2013–2016 celebration periods; Table S6. The meteorological average values in Wuhan from 2013 to 2016; Table S7. The PM_2.5_ concentration differences and meteorological average values in Chengdu from 2013 to 2016; Table S8. Meteorological data of Zhengzhou on New Year’s Eve and the following two days in 2016.
