# Supplementary material for: The Effects of Fireworks Discharge on Atmospheric PM2.5 Concentration in the Chinese Lunar New Year
Source: Int J Environ Res Public Health. 2020 Dec 13;17(24):9333. doi: 10.3390/ijerph17249333 (PMC7764231; doi:10.3390/ijerph17249333)
Supplement: Supplementary file 1 [file ijerph-17-09333-s001.pdf]

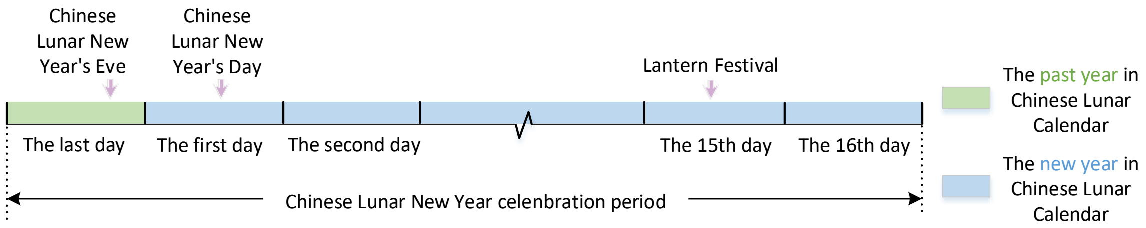

Figure S1. Scheme of important timing during the Chinese Lunar New Year celebration period.

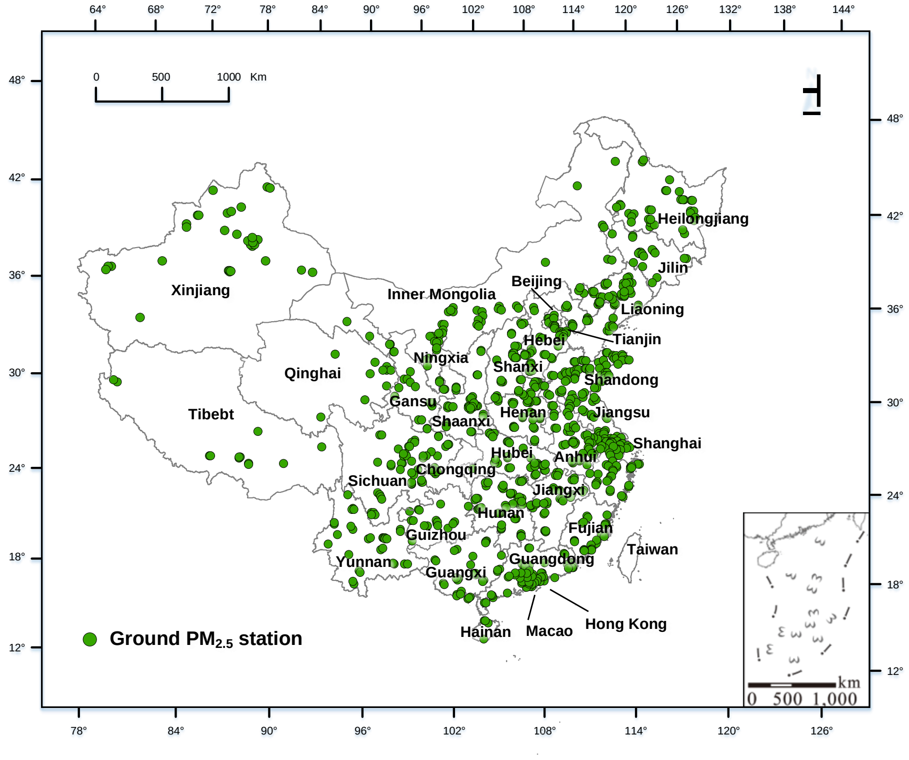

Figure S2. Distribution of PM2.5 monitoring stations in mainland China by the end of 2016. Data and site information for Hong Kong, Macao, and Taiwan are not available for now.

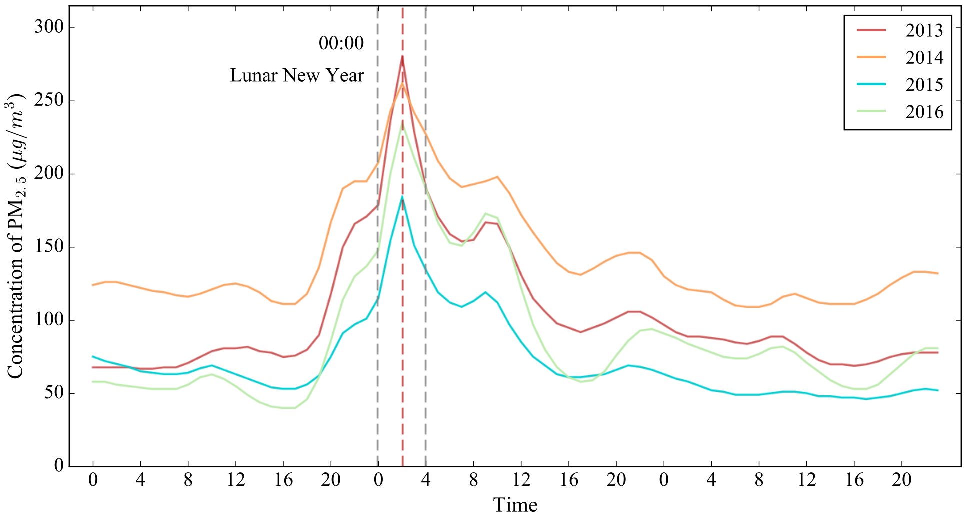

Figure S3. National hourly $PM_{2.5}$ concentration during New Year's Eve and the next two days. The dashed red line represents 02:00 on New Year's Day, and the dashed gray lines represent 0:00 and 04:00 on New Year's Day, respectively.

Parameter	Code name	Unit	Height of data acquisition
Temperature	TEMP	K	Specified height above ground/2 m[a]
Relative humidity	RH	%	Specified height above ground/2 m
Wind speed	WS（U-wind/ V-wind）	m/s	Specified height above ground/10 m[a]
Height of planetary boundary layer	HPBL	m	Ground or water surface
Surface pressure	SP	Pa	Ground or water surface

Table S1. Meteorological data information.

The data format changed from Grib1 to Grib2 after 6 December 2007, and [a] indicates the further supplement of measurement height information in the Grib2 format from 14 January 2015. After updating, the 2 m and 10 m measurements of temperature and wind speed data were used, respectively.

Year	2002	2003	2004	2005	2006
Data of Chinese New Year	12 Feb	1 Feb	22 Feb	9 Feb	29 Jan
Year	2007	2008	2009	2010	2011
Data of Chinese New Year	18 Feb	7 Feb	26 Jan	14 Feb	3 Feb
Year	2012	2013	2014	2015	2016
Data of Chinese New Year	23 Jan	10 Feb	31 Jan	19 Feb	8 Feb

Table S2. Chinese New Year dates in 2002-2016.

Year	New Year's Eve	New Year's Day	Lantern Festival	The day after Lantern Festival (The end of celebration period)
2013	9 Feb	10 Feb	24 Feb	25 Feb
2014	30 Jan	31 Jan	14 Feb	15 Feb
2015	18 Feb	19 Feb	5 Mar	6 Mar

2016	7 Feb	8 Feb	22 Feb	23 Feb

Table S3. Dates of important days during 2013-2016 Chinese Lunar New Year celebration periods.

Year	Tibetan New Year	Firework display time	Within Chinese Lunar New Year celebration period or not
2013	10 Feb	8 Feb	No
2014	2 Mar	28 Feb	No
2015	19 Feb	17 Feb	No
2016	9 Feb	7 Feb	Yes, same date

Table S4. Tibetan New Year date and its firework display time.

The 55 ethnic minorities and the Han nationality comprise the 56 ethnic groups in China, and each ethnic minority has its own festival. The Tibetan people are one of the ethnic minorities. Tibetans are the main population group in the Tibet area, and other minorities and Han people are relative fewer in this region. In Tibetan custom, on the 29th day of the past year in the Tibetan calendar, people will discharge fireworks to celebrate New Year, too. According to the date of the Tibetan New Year and the Lunar New Year, the 2013-2015 Tibetan New Year firework displays were not in the Lunar New Year celebration period. The 2016 Tibetan New Year firework displays were the same time as the Lunar New Year. It can therefore be seen that the firework displays for the Tibetan New Year will not alter the result of the city-level $PM_{2.5}$ response classification.

Year	***Significant***-response city	***Obvious***-response city	***Weak***-response city
2013	24	5	2
2014	20	5	6
2015	17	8	6
2016	19	4	8

Table S5. Classification results of $PM_{2.5}$ response in 31 provincial capitals during 2013-2016 celebration periods.

	2013	2014	2015	2016
Wind speed (0.1 m/s)	31	39	34	33
Precipitation (0.1mm)	1.3	0.2	2.3	0

Table S6. The meteorological average values in Wuhan from 2013 to 2016.

The meteorological data values are the average data of the corresponding data on Chinese New Year's Day and the two days before and after the New Year's Day.

	2013	2014	2015	2016
$PM_{2.5}$ concentration (μg/m^3)	189	211	110	120
Wind speed (0.1 m/s)	12	10	17	9
Precipitation (0.1mm)	2.0	0	0	0

Table S7. The $PM_{2.5}$ concentration differences and meteorological average values in Chengdu from 2013 to 2016.

The differences in PM2.5 concentrations are the differences between the maximum hourly values on Chinese New Year's Day and the average values of the two days before and after the corresponding New Year's Day. The meteorological data values are the average data of the corresponding data on Chinese New Year's Day and the two days before and after the New Year's Day.

	Wind speed (0.1 m/s)	Precipitation (0.1 mm)	Relative humidity (1 %)	Temperature (0.1 °C)	Surface pressure (0.1 hPa)
7 Feb	45	0	24	31	9481
8 Feb	32	0	25.5	75	9437
9 Feb	23	0	27.5	68	9446

Table S8. Meteorological data of Zhengzhou on New Year's Eve and the following two days in 2016.
